# The Evolution and Maturation of Teams in Organizations: Convergent Trends in the New Dynamic Science of Teams

**DOI:** 10.3389/fpsyg.2020.02128

**Published:** 2020-09-04

**Authors:** Marissa L. Shuffler, Eduardo Salas, Michael A. Rosen

**Affiliations:** ^1^College of Behavioral Social and Health Sciences, Clemson University, Clemson, SC, United States; ^2^Department of Psychology, Rice University, Houston, TX, United States; ^3^Department of Anesthesiology and Critical Care Medicine, Johns Hopkins University School of Medicine, Baltimore, MD, United States

**Keywords:** teams and groups, time, teamwork, work team, team training

Teams play a central role in the most innovative (Ahmadpoor and Jones, 2019), safety critical (Salas et al., 2020), and economically impactful work (Duhigg, 2016). They pervade modern organizations and drive performance outcomes (LePine et al., 2008; Hughes et al., 2016) and worker well-being (Welp and Manser, 2016; Mathieu et al., 2017). Consequently, researchers across a broad array of disciplines have focused on teams as an object of inquiry. Understanding and improving team functioning is a complex multi-level scientific problem, and, over the decades, much has been learned (Salas et al., 2018). The science of teams comprises a broad and deep knowledge base of both theory and empirical evidence, including topics such as structural inputs to team performance (e.g., team member composition, organizational context, effects of technology) and team interaction processes and emergent states (e.g., leadership, communication, mutual trust, collective efficacy). However, while the science of teams is strong, much remains to be discovered–especially from a temporal perspective. Calls for dynamic views of teams are not new (Cronin et al., 2011), but the field is shifting in numerous theoretical and methodological ways. The confluence of driving forces magnifying in intensity (i.e., modern work becoming more collaborative) and restraining forces reducing in intensity (i.e., traditional, resource-intensive measurement methods giving way to new unobtrusive, embedded metrics) allows for the science of teams to explore new directions in the dynamics of teams.

This new phase of team science is concerned with the *temporality of teams*: how teams evolve and mature, and how team dynamics play out over time. Accordingly, the purpose of this special issue is to offer current theory and research that describes the state of temporality in team science thus far, identifies future research needs, and highlight impactful insights for practice. The articles in this special issue represent work across the broad spectrum of research incorporating time in new ways. In this commentary, we identify eight themes in dynamic approaches to teams, and highlight how articles in this special issue exemplify these trends in the field (See Table 1). More specifically, we note that dynamics are impacting the fundamental theory and methods of the science of teams (Themes 1–4), the types of team phenomena being investigated (Themes 5–6), and application of team science in context (Theme 7) and to interventions that promote team effectiveness (Theme 8).

**Table 1:** Overarching themes across the special issue regarding temporality and the science of teams.

**Theme**	**Description and implications for the science of teams**
**Theory and methods for advancing a dynamic science of teams**	
1. Methods and theories of team dynamics are co-evolving, moving through a phase of discovery and diversity toward a more robust and stable set of conceptual and analytic tools	•New measurement methods are enabling the development and application of new theoretical frameworks •Methodological practices vary widely in current research evaluating team dynamics •Best practices are beginning to emerge including guidance linking methods to dynamic team phenomena
2. The science of teams is becoming multi (time) scale, not just multi-level, and future research will investigate phenomena across very short and very long timescales	•Research is pursuing an understanding of team member interactions that play out at very short timescales •Research is pursuing team functioning over long time scales •Future research will begin to understand how phenomena operating at one time scale influence those operating at much different time scales
3. Team research is revisiting traditionally static or stable characteristics of teams, and future research will better characterize how those characteristics or their effects change over time	•Traditionally, team inputs have been viewed as static, or time invariant •Many of the traditional team inputs or their effects on team interactions and outcomes have been shown to change over time •Team science will incorporate an understanding of how these factors or their effects change over time
4. The science of teams incorporates ever broader ranges of individual factors and will routinely include the biological and physiological dynamics of team members	•Biological and physiological phenomena are being included in models and studies of team functioning •This research area will advance quickly due to parallel advances in other areas of social and organizational sciences
**Dynamic team phenomena being researched**	
5. Learning in teams is critical in modern organizations, and the science of teams is uncovering the dynamics of team learning processes and the context of learning	•Learning is inherently temporal •Research is refining a dynamic understanding of team learning processes as well as how the context of learning emerges and changes over time
6. Emotions are central to teams, and the science of teams is uncovering the dynamics of the ebb, flow, and mutual influence of affect amongst team members	•Emotions of team members rise and fall, and influence one another in complex ways •Research is beginning to incorporate dynamic views and methods of affect measurement into studies •A more dynamic understanding of team affect can inform many research topics, including a wide range of team interpersonal processes
**Context of research, generalizability, and interventions from a dynamic science of teams**	
7. High risk/high stake industries are leading the way for team dynamics research, but the science of teams must attend to generalizability across settings	•Much of the research on team dynamics is coming from a limited set of organizational settings •Empirical studies of team functioning may be more context bound than less temporally focused studies •The science of teams will need to establish methods and practices for establishing generalizability of highly dynamic models and studies
8. A better understanding of team dynamics can drive new and adaptive interventions to shape dynamics	•Traditional methods of team development are being revisited given the emerging science of team dynamics •New, dynamic, and adaptive interventions will emerge, both from the research community and from practice

## Methods and Theories of Team Dynamics are Co-evolving Toward a More Robust Set of Conceptual and Analytic Tools

New theories require new measurement methods and new methods enable different conceptualizations of team dynamics. This co-evolution of method and theory is currently underway and involves both advances in data acquisition and analysis (Rosen et al., 2015). For example, systems dynamics, and more broadly complexity science, has long been an inspiration to theory development for team and group researchers (Arrow et al., 2000). In this issue, Meinecke et al. (2019) elaborate on this theoretical lens and describe the application of state-space grids to the challenges of operationalizing systems dynamics concepts for measuring and understanding team dynamics. Relatedly, Marques-Quinteiro et al. (2019) use a complex adaptive systems perspective and latent growth modeling to explore the interplay of behavior and affect in teams. These studies employ conceptually similar frameworks and disparate methods to explore important teamwork issues. This diversity is healthy for the field. However, as the measurement and analytic toolbox grows, it is important to codify what is known about what methods are appropriate for which team phenomenon and under which conditions. Delice et al. (2019) provide a valuable framework for mapping methodological choices to facets of team dynamics. As Kolbe and Boos (2019) clearly articulate, historically, methods that meaningfully capture team dynamics tend to be more labor intensive than those that measure team phenomena at a much lower temporal resolution. For example, communication coding at the utterance level requires far more researcher time than summative ratings of communication, and even current implementations of automated methods are more complicated and effortful to conduct than survey research. However, methods are advancing quickly. We foresee more and better methodological options in coming years, which will allow for and require new ways of theorizing about teams.

## The Science of Teams Is Becoming Multi (Time) Scale, not Just Multi-level

Pursuing a dynamic approach to teams requires decisions about how time is conceptualized and operationalized (Mohammed et al., 2009). Conceptually, how is time being incorporated into theory and hypotheses? Operationally, decisions need to be made about appropriate temporal granularity or resolution for measurement, and how this supports the valid measurement of different phenomena. This includes thinking longitudinally about teams existing and changing over very long periods of time [e.g., research on teams on long duration space exploration missions, (Bell et al., 2019); or functioning in other Isolated, Confined and Extreme (ICE) environments; (Landon et al., 2019)], as well as looking at very “thin slices” of interaction across multiple streams of data (i.e., linguistic and paralinguistic communication, physiological activation, behavior; Rosen et al., 2018). There is exciting work in each of these ranges of timescales for team dynamics; however, there is very little that integrates them both. From research on interpersonal dynamics outside of work team settings we know that patterns on one time scale (e.g., seconds to milliseconds) can predict patterns over very different timescales [e.g., years; (Gottman et al., 2002)]. To progress, the field needs more cross-timescale studies, refined methods for conducting such analyses, and conceptual tools for building multi-scale (not just multi-level) theory. Future research will investigate phenomena across differing (i.e., short, long) time scales.

## Team Science Is Revisiting Traditionally Static or Stable Team Characteristics

Everything changes. So, exactly how stable are team inputs? Does their relationship to team dynamics and outcomes change over time? These questions drive important research in team dynamics focused on better understanding stability and change in teams (Kerrissey et al., 2020). First, research is elucidating how the relationship between inputs and team dynamics or outcomes shifts as a function of time. For example, Burke et al. (2019) investigate how the instrumentality of different team roles changes over extended team missions. Second, research is revisiting how aspects of teams traditionally viewed as stable and unchanging through a team performance episode, do in fact change and how this relates to outcomes. Bedwell (2019) explores how membership fluidity impacts shared mental model development. As described by Benishek and Lazzara (2019), our understanding of these and other team attributes once conceived of as time invariant will be reevaluated and allow us to refine what we thought were stable team attributes. Undoubtedly, future research will better extrapolate how team characteristics and their effects change over time.

## Team Science now Incorporates Ever Broader Ranges of Individual Factors to Include the Biological and Physiological Dynamics of Team Members

The science of teams has pursued multi-level approaches for decades (Klein and Kozlowski, 2000); however, the strata continue to deepen. It is no longer just individuals nested in teams, but biological attributes and physiological processes nested within individuals within teams within larger organizational entities and time. Landon et al. (2019) provide a wide ranging and integrative review of the topic as it relates to performance within ICE settings, and Stevens et al. (2019) provide a remarkable example of how patterns of physiological activation across team members can be identified and used to predict team outcomes. These articles are exemplars of the emerging area of team physiological dynamics, which has accelerated rapidly in recent years (Kazi et al., 2019). The science of teams can progress quickly in this area by exploring related areas of social (Cacioppo et al., 2000) and organizational neuroscience (Becker et al., 2011) and interpersonal physiological dynamics outside of work team contexts (Palumbo et al., 2017). The rapid improvement in wearable physiological measurement devices make the collection of this type of data increasingly feasible, even in field settings. Consequently, physiological measurement in team studies will become increasingly common, and methods and theory will mature rapidly. As this work matures, measurement and theory development will have to address linkages between these lower level biological states and processes, and higher level, abstract constructs such as mutual trust and support or other team processes and emergent states. Innovative approaches to handling these issues have been introduced (Luciano et al., 2018), but much more remains to be done.

## The Science of Teams Is Uncovering the Dynamics of Team Learning Processes and the Impact of Context on Learning

Demands for continuous improvement are commonplace in today's organizations [e.g., Toussaint and Ehrlich (2017)]. Market competition is frequently steep, and external and internal environments shift [e.g., Autor et al. (2016)]. To succeed, teams need to learn from their experiences and the experiences of others. Consequently, team learning has emerged as both a critical team process and a type of performance investigated from a dynamic perspective. Given that learning inherently involves change, time is central to team learning. Wiese and Burke (2019) critically review extant team learning research and formulate a temporal model of how team learning unfolds over time. In addition to the learning process itself, the local conditions within the team and its context influence if or how learning happens. This learning climate has historically been viewed as a relatively stable or slow-moving phenomenon. However, Harvey et al. (2019) apply systems dynamics modeling to forward a theory of team learning climate, and how it rises and falls with changes in levels of psychological safety, cohesion, efficacy, and goal orientation within the team. Again, several of these constructs previously considered time invariant can be reexamined through a dynamic lens to move the field forward.

## The Science of Teams Is Uncovering The Dynamics of the EBB, Flow, and Mutual Influence of Affect Amongst Team Members

Affect is not a novel concept to the science of teams; in fact, emotions are central to effective teamwork (Salas et al., 2018). The roles of trust, cohesion, collective orientation and numerous other attitudes, emotional states and dispositional variables on team effectiveness have been widely researched. However, this new dynamic-focused approach to teams allows for a more nuanced understanding of how affect changes over time, how it influences and is influenced by other team phenomena over time, and how the effect of team members is shared.

In our special issue, the dynamic nature of affect is explored using similar methodological approaches, yet with two very different sets of constructs in order to expand our understanding of how teams may grow and change in their affective states over time. Woodley et al. (2019) utilize latent growth and consensus emergence modeling techniques to investigate changes in team potency over time. Marques-Quinteiro et al. (2019) also apply latent growth modeling, but to cohesion and its relationship to coordination and performance. Advances in understanding the dynamics of affect in teams can help to address a wide range of issues in the study of team functioning, from stress, burnout, and well-being, to conflict management and relationship building. While not limited to interpersonal team processes, a more dynamic understanding of affect in teams can certainly advance this critical aspect of teams.

## High Risk/High Stake Industries Are Leading the Way for Team Dynamics Research, but the Science of Teams Must Attend to Generalizability Across Settings

Context matters, and certain industries have embraced the importance of team dynamics as the links between team functioning and valued organizational outcomes in that industry are particularly salient. Articles in this special address spaceflight (Bell et al., 2019; Pendergraft et al., 2019) military (Demir et al., 2019; Johnston et al., 2019), healthcare (Stevens et al., 2019), and isolated and confined environments (Landon et al., 2019). While this list of industries is by no means exhaustive of those pursuing team-based work strategies or engaged in research efforts to understand and improve team dynamics, it is representative of the key contributors. Advancing the science of teams through dynamic approaches can add detail and specificity to the models (e.g., higher granularity of measurements) and many of the theories and analytic approaches applied to date emphasize principles such as sensitivity to initial conditions, all of which suggest that more dynamic models may be more tightly bound to their context. The maturation of dynamic approaches to the science of teams requires parallel developments in how research handles context in studies, specifically the role context plays constraining and enabling the occurrence and meaning of different team dynamics (Johns, 2006). Better methods for representing and interpreting context will be crucially important to a robust science of team dynamics.

## A Better Understanding of Team Dynamics can Drive New and Adaptive Interventions for Team Effectiveness

As a practical matter, a more refined understanding of team dynamics is valuable to organizations only if it can be translated into mechanisms for improved performance. A more robust understanding of how team dynamics drive performance outcomes enables new and improved interventions to support effective team dynamics. This includes advancing our knowledge about how to most effectively use familiar interventions like meetings (Mroz et al., 2019) and team training (Johnston et al., 2019), as well as more novel approaches like automated feedback in virtual teams (Glikson et al., 2019) and well-being interventions (Wiese and Burke, 2019). The future will continue to see extension and refinement of tried and true methods of team development informed by more dynamic understanding of teams as well as new forms of real-time support for teams and use of synthetic agents as team members and coaches (Demir et al., 2019). As is often the case, practice may lead research in the area of intervention development. Researchers should look to innovations in the field and capitalize on them to generate insights into underlying mechanisms of team dynamics.

## Conclusion

The time has arrived for a serious treatment of time in all aspects of research on teams. The need for dynamic approaches to understanding teams has long been heralded. The articles in this special issue demonstrate that the field is delivering on that vision of research on teams, a vision that places temporality at the center of both theory, methods, and evidence-driven applications. We are at the leading edge of this transformation of the field. Theory is still nascent for team phenomenon over and across very long or very short timescales. Methodological practices are in a divergent, exploratory phase where wide variation of new methods is observed and best practices have yet to emerge. But the progress over recent years is remarkable, and the value of pursuing a science of team temporality is clear.

## Author Contributions

All authors were involved in drafting and reviewing final manuscript.

## Conflict of Interest

The authors declare that the research was conducted in the absence of any commercial or financial relationships that could be construed as a potential conflict of interest.
